# Bone fracture is associated with incident myocardial infarction in long-term follow-up

**DOI:** 10.1186/s12889-024-18897-w

**Published:** 2024-05-23

**Authors:** Mei-Liang Zheng, Xiang-Peng Du, Xin-Chun Yang, Mei-Li Zheng

**Affiliations:** 1 Heart Center, Beijing Chao-Yang Hospital, Capital Medical University, 8# Gong-Ti South Road, Beijing, China; 2 Beijing Key Laboratory of Hypertension Research, Beijing, China; 3 Department of Orthopedics, The Second Central Hospital of Baoding, Zhuozhou, Hebei China; 4 Department of Cardiology, Weihaiwei People’s Hospital, Weihai, Shandong China

**Keywords:** Bone fracture, Myocardial infarction, CHNS

## Abstract

**Background:**

The association between bone fracture and cardiovascular diseases is examined in this study. While basic research has established a connection between fractures and heart attacks through the linkage between bones and arteries, population studies have not provided clear evidence. The aim of the present study is to investigate the association between bone fracture and the occurrence of myocardial infarction in a natural population during long-term follow-up.

**Methods:**

A total of 13,196 adult participants with bone fracture history at baseline from the China Health and Nutrition Survey (CHNS) prospective cohort were included in this study. Baseline investigation was performed in 1997–2009 and the outcome was followed up till 2015. Hazard ratios (HRs) and their corresponding 95% confidence intervals (CIs) were calculated using Cox proportional hazards models.

**Results:**

From 1997 to 2015, a total of 329 incident myocardial infarction cases were identified. In univariate and multivariate Cox regression analysis, a history of bone fracture was associated with an increased risk of myocardial infarction incidence in the total population (for the crude model: HR = 2.56, 95% CI 1.83–3.53, *P* < 0.001; for the multivariate model: HR = 1.43, 95% CI 1.02–1.99, *P* = 0.036). In the stratified analysis, bone fracture was not associated with an increased risk of incident myocardial infarction in subjects with age < 50 years (HR = 0.71, 95% CI 0.34–1.47, *P* = 0.356), but significantly associated with an increased risk of incident myocardial infarction in subjects with age ≥ 50 years (HR = 1.80, 95% CI 1.23–2.63, *P* = 0.003).

**Conclusions:**

It is suggested by the present study that bone fracture may be associated with an increased risk of incident myocardial infarction in the elderly population during long-term follow-up.

## Background

Myocardial infarction, globally, stands as the foremost cause of mortality. Although traditionally linked to aging, it has increasingly afflicted younger individuals in recent times. The global incidence of myocardial infarction ranges from 1.5 to 2.3 per 1000 person-years, with a particularly notable increase among the young and middle-aged population [1–5]. Similarly, bone fracture poses a significant burden on healthcare systems. A recent Chinese study reported clinical fracture prevalence of 4.1% in men over 40, 4.0% in men over 80, 4.2% in women over 40, and 4.5% in women over 80 years of age [6]. Similar to myocardial infarction, osteoporotic fractures represent another age-related ailment, their occurrence rising with age [6–8].

While bone fracture and myocardial infarction are primarily associated with distinct organ systems, they share a connection through the interplay between bones and blood vessels. Fundamental research has shown that arterial calcification constitutes a pivotal pathophysiological mechanism in myocardial infarction, closely linked to atherosclerotic plaque buildup and instability [9], thereby forecasting adverse arterial events, including myocardial infarction [10, 11]. Numerous animal studies have established that osteoporosis and arterial calcification share common molecular pathways [12, 13]. Particularly, smooth muscle cells surrounding blood vessels have the potential to undergo transformation into osteo/chondrogenic cells during arterial calcification [14, 15]. The aging process and chronic kidney disease can induce bone loss, leading to osteoporosis [16, 17], and arterial calcification, resulting in arterial rigidity and plaque instability [18, 19]. Population studies have identified an association between arterial calcification and reduced bone mineral density (even after adjusting for age) [20]. Additionally, correlations have been observed between lower lumbar volumetric bone mineral density and greater coronary artery calcium scores in both women and men, as well as greater abdominal aortic calcium scores in the Multi-Ethnic Study of Atherosclerosis [21]. A case-control study [22], focusing on hip fracture patients matched with those without hip fractures, independently established that hip fractures were associated with an increased risk of subsequent myocardial infarction during follow-up. Another retrospective study [23] demonstrated a correlation between vertebral fractures and myocardial infarction in hemodialysis patients.

Fundamental research has substantiated the potential association between fractures and myocardial infarction through age-related osteoporosis and arterial calcification. Population studies have hinted at this link in high-risk groups, likely due to the relatively low incidence of myocardial infarction in the general population. Nevertheless, research in natural populations is still imperative. In the current study, we have included adult participants from the China Health and Nutrition Survey (CHNS) prospective cohort and have scrutinized the relationship between bone fracture and the occurrence of myocardial infarction in this natural population during long-term follow-up.

## Methods

### Study population

**Fig. 1:**
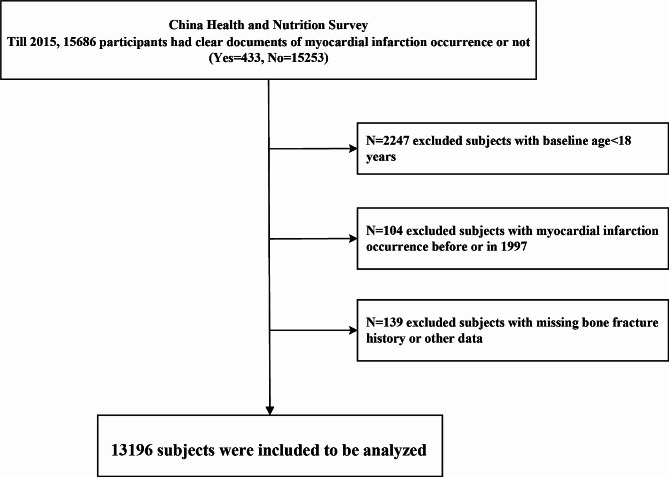
Flowchart depicting the analysis sample

This study utilized longitudinal data from the population-based China Health and Nutrition Survey (CHNS) [24]. The CHNS is an ongoing prospective cohort study comprising over 30,000 participants across China. It is a nationally representative survey that commenced in 1989 and has completed ten survey rounds up to 2015. Detailed survey procedures have been previously documented [25, 26]. Information regarding bone fracture and myocardial infarction was recorded starting from 1997. We analyzed the association between bone fracture and incident myocardial infarction in participants aged 18 years and older during the baseline investigation in surveys 1997–2009 and followed up the outcome till survey 2015. Participants who experienced myocardial infarction before the baseline, were under 18 years of age, or had missing fracture history were excluded (Fig. 1). The research protocol received approval from the Ethics Committee of Beijing Chao-Yang Hospital Affiliated with Capital Medical University in China.

### Covariates

Demographic and lifestyle information of participants was gathered through questionnaires, encompassing birth year, gender, medical history, smoking habits, and alcohol consumption. This study was conducted in five different periods, corresponding to five distinct baseline years. Baseline age was determined as the difference between the baseline year and the birth year, with participants under 18 years of age being excluded. Baseline history of hypertension, diabetes, smoking, and alcohol consumption were derived from records before and during the baseline visit. Body mass index (BMI) was computed as the ratio of weight in kilograms to the square of height in meters. Skilled investigators measured body weight and height using standardized measurement techniques.

In 2009, 9549 subjects underwent blood biomarker assessments, including total cholesterol (TC), low-density lipoprotein cholesterol (LDL-C), serum creatinine (SCr), among others. For further analysis, we included TC, LDL-C, and estimated glomerular filtration rate (eGFR) to account for the influences of dyslipidemia and kidney function decline. eGFR was calculated using the 2009 Chronic Kidney Disease Epidemiology Collaboration (CKD-EPI) equation [27]: For females, if SCr is ≤ 0.7 mg/dl, eGFR equals 144×(Cr/0.7) ^−0.329^ × (0.993) ^age^, and if SCr is > 0.7 mg/dl, eGFR equals 144×(Cr/0.7) ^−1.209^ × (0.993) ^age^. For males, if SCr is ≤ 0.9 mg/dl, eGFR equals 141×(Cr/0.9) ^−0.411^ × (0.993) ^age^, and if SCr is > 0.9 mg/dl, eGFR equals 141×(Cr/0.9) ^−1.209^ × (0.993) ^age^. These variables were included as both continuous and categorical variables (TC was categorized at 5.2 mmol/L [TC: <5.2 and ≥ 5.2 mmol/L], LDL-C at 3.4 mmol/L [LDL-C: <3.4 and ≥ 3.4 mmol/L], and eGFR at 90 ml/min/1.73 m² [eGFR: <90 and ≥ 90 ml/min/1.73m^2^]).

### Primary exposure and outcome

Bone fracture was defined as a self-reported fracture history. Myocardial infarction was defined as a self-reported diagnosis of myocardial infarction, and the time scale was calculated using baseline year and self-reported incident year of myocardial infarction, for those self-reported incident year missing (< 5%), we filled with the year of first self-reported diagnosis of myocardial infarction. Myocardial infarction served as the primary outcome in this study, with data recorded from the baseline year (not included) up to 2015. Specifically, among the participants, a total of 433 participants experienced myocardial infarction, and 104 occurred prior to 1997 (Fig. 1).

### Statistical analyses

All statistical analyses were carried out using R statistical software version 4.2.0, and graphs were generated using GraphPad Prism 6 (GraphPad Software, Inc.). Continuous variables were presented as mean ± standard deviation (SD), while categorical variables were expressed as counts and percentages. Univariate and multivariate Cox regression analyses were employed to assess the relationships between myocardial infarction and bone fracture, along with other variables. The interaction terms between fractures and other covariables were also evaluated. All statistical tests were two-tailed, and P-values less than 0.05 were considered statistically significant.

## Results

**Fig. 2:**
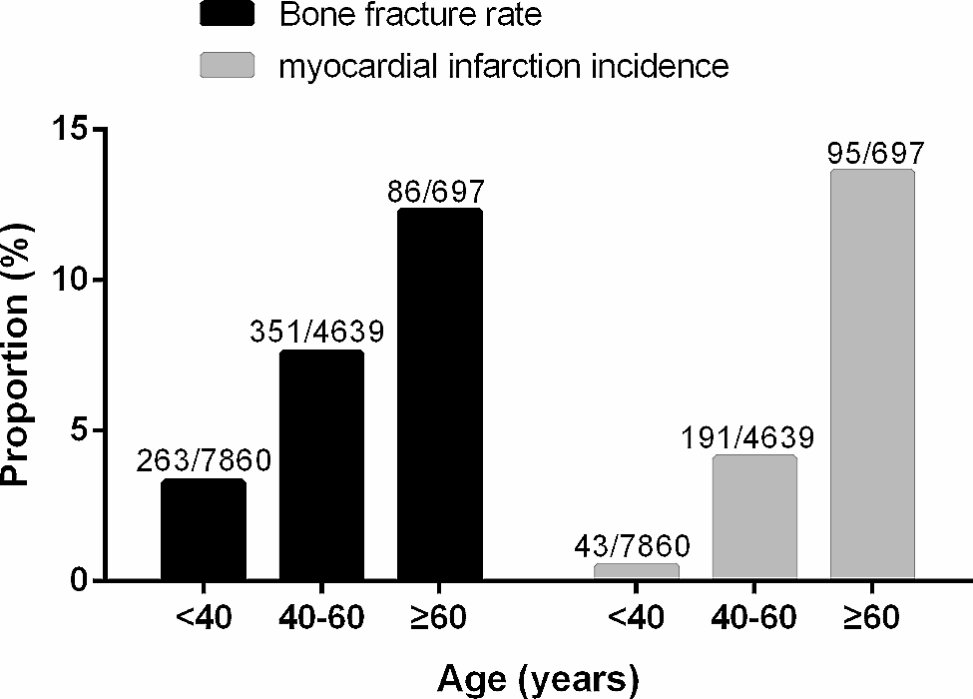
Bone fracture rates at baseline and myocardial infarction incidences in different age groups (numbers of fractures or incident myocardial infarction/total are displayed)

The baseline characteristics of the participants are displayed in Table 1, both overall and stratified by bone fracture status. Overall, the participants were at the average of 36.0 years old when they were enrolled in the study, with average BMI of 23.1 kg/m^2^, 8.3% having hypertension, 2.0% having diabetes, 47.8% being males, 23.9% being smokers, and 30.5% being alcohol drinkers. Participants with bone fracture history tended to have higher myocardial infarction incidence, age and BMI, and were more likely to be male, have hypertension, diabetes, smoking and drinking habits. The estimated annual incidence rate is 149, 353, and 138 cases per 100,000 individuals for the total participants, participants with bone fracture, and participants without bone fracture. With increasing age, both the fracture rate and myocardial infarction incidence exhibited significant increases. Participants were categorized into three age groups: <40, 40–60, and ≥ 60 years. In these groups, fracture rates were 3.3%, 7.6%, and 12.3% (*P* < 0.001), while myocardial infarction incidence was 0.5%, 4.1%, and 13.6% (*P* < 0.001) (Fig. 2).

**Table 1:** Characteristics of the study population

	Overall (*N* = 13,196)	Bone fracture		*P* value
		Yes (*N* = 700)	No (*N* = 12,496)	
Estimated annual incidence of myocardial infarction, cases/per 100,000 people	149	353	138	-
Myocardial infarction incidence, n (%)	329 (2.5)	42 (6.0)	287 (2.3)	< 0.001
Age, years	36.0 ± 13.4	43.5 ± 13.5	35.6 ± 13.2	< 0.001
Age categories, years				< 0.001
< 50	10,904 (82.6)	467 (66.7)	10,437 (83.5)	
≥ 50	2292 (17.4)	233 (33.3)	2059 (16.5)	
Gender, n (%)				< 0.001
Male	6308 (47.8)	399 (57.0)	5909 (47.3)	
Female	6888 (52.2)	301 (43.0)	6587 (52.7)	
BMI, kg/m^2^	23.1 ± 3.9	22.7 ± 3.4	23.1 ± 3.9	0.002
BMI categories, kg/m^2^				0.059
< 24	8468 (64.2)	473 (67.6)	7995 (64.0)	
≥ 24	4728 (35.8)	227 (32.4)	4501 (36.0)	
Hypertension history, n (%)				< 0.001
Yes	1099 (8.3)	162 (23.1)	937 (7.5)	
No	12,097 (91.7)	538 (76.9)	11,559 (92.5)	
Diabetes history, n (%)				< 0.001
Yes	258 (2.0)	35 (5.0)	223 (1.8)	
No	12,938 (98.0)	665 (95.0)	12,273 (98.2)	
Smoking (ever), n (%)				< 0.001
Yes	3160 (23.9)	353 (50.4)	2807 (22.5)	
No	10,036 (76.1)	347 (49.6)	9689 (77.5)	
Alcohol consumption (ever), n (%)				< 0.001
Yes	4027 (30.5)	427 (61.0)	3600 (28.8)	
No	9169 (69.5)	273 (39.0)	8896 (71.2)	

BMI, body mass index

In univariate Cox regression analysis, a history of bone fracture was significantly associated with an increased risk of incident myocardial infarction (HR = 2.56, 95% CI 1.83–3.53, *P* < 0.001). After adjusting for age, gender, BMI, hypertension, diabetes, smoking, and alcohol consumption, a history of bone fracture remained significantly associated with an increased risk of incident myocardial infarction (HR = 1.43, 95% CI 1.02–1.99, *P* = 0.036) (Table 2). In the stratified analysis, a history of bone fracture was not associated with an increased risk of incident myocardial infarction in subjects with age < 50 years (HR = 0.71, 95% CI 0.34–1.47, *P* = 0.356), but significantly associated with an increased risk of incident myocardial infarction in subjects with age ≥ 50 years (HR = 1.80, 95% CI 1.23–2.63, *P* = 0.003), and the interaction between fractures and age was also significant (P for interaction < 0.001); a history of bone fracture was also significantly associated with an increased risk of incident myocardial infarction in subjects with BMI < 24 kg/m^2^ (HR = 1.62, 95% CI 1.03–2.55, *P* = 0.035), subjects without diabetes (HR = 1.44, 95% CI 1.01–2.05, *P* = 0.044), non-smokers (HR = 1.66, 95% CI 1.09–2.51, *P* = 0.018), non-drinkers (HR = 1.76, 95% CI 1.12–2.76, *P* = 0.015) (Table 3).

**Table 2:** Univariate and multivariate Cox regression analysis of bone fracture on incident myocardial infarction

	HR	95%CI	*P* value
Crude	2.56	1.85–3.53	< 0.001
Adjusted for age	1.55	1.12–2.14	0.009
Adjusted for age, gender	1.54	1.11–2.14	0.009
Adjusted for age, gender, BMI	1.55	1.12–2.15	0.008
Adjusted for age, gender, BMI, hypertension	1.43	1.03–1.99	0.033
Adjusted for age, gender, BMI, hypertension, diabetes	1.43	1.03–1.98	0.035
Adjusted for age, gender, BMI, hypertension, diabetes, smoking	1.42	1.02–1.99	0.037
Adjusted for age, gender, BMI, hypertension, diabetes, smoking, alcohol consumption	1.43	1.02–1.99	0.036

BMI, body mass index

**Table 3:** Stratified analysis of bone fracture on incident myocardial infarction

	Events/Total	Crude				Multivariate*			
		HR	95%CI	*P* value		HR	95%CI	*P* value	*P* value for interaction
Age categories (years)									
< 50	143/10,904	1.26	0.62–2.57	0.524		0.71	0.34–1.47	0.356	
≥ 50	186/2292	2.06	1.42–2.99	< 0.001		1.80	1.23–2.63	0.003	< 0.001
Gender									
Male	165/6308	1.90	1.18–3.07	0.008		1.33	0.81–2.18	0.261	
Female	164/6888	3.45	2.22–5.36	< 0.001		1.46	0.93–2.30	0.104	0.544
BMI categories (kg/m^2^)									
< 24	164/8468	2.66	1.71–4.13	< 0.001		1.62	1.03–2.55	0.035	
≥ 24	165/4728	2.57	1.60–4.15	< 0.001		1.21	0.74–1.98	0.455	0.956
Hypertension history									
Yes	90/1099	1.71	1.04–2.81	0.035		1.50	0.91–2.48	0.111	
No	239/12,097	2.12	1.37–3.29	< 0.001		1.32	0.84–2.06	0.231	0.885
Diabetes history									
Yes	21/258	2.07	0.76–5.65	0.156		1.62	0.57–4.61	0.370	
No	308/12,938	2.47	1.75–3.48	< 0.001		1.44	1.01–2.05	0.044	0.851
Smoking									
Yes	103/3160	1.35	0.78–2.34	0.277		1.09	0.63–1.89	0.756	
No	226/10,036	3.75	2.51–5.61	< 0.001		1.66	1.09–2.51	0.018	0.113
alcohol consumption									
Yes	122/4027	1.57	0.96–2.56	0.071		1.12	0.68–1.83	0.651	
No	207/9169	4.00	2.60–6.18	< 0.001		1.76	1.12–2.76	0.015	0.157

*Multivariate model included fracture, age, gender, BMI, hypertension, diabetes, smoking (ever), alcohol consumption (ever). BMI, body mass index

To account for the influences of dyslipidemia and kidney function decline, we included TC, LDL-C, and eGFR data from the 2009 blood marker test. These variables were incorporated as either continuous or categorical variables (TC: <5.2 and ≥ 5.2 mmol/L; LDL-C: <3.4 and ≥ 3.4 mmol/L; eGFR: <90 and ≥ 90 ml/min/1.73 m²). After adjusting for all covariates, including age, gender, BMI, hypertension, diabetes, smoking, alcohol consumption, TC, LDL-C, and eGFR, a history of bone fracture remained significantly associated with an increased risk of incident myocardial infarction (HR = 1.94, 95% CI 1.12–3.33, *P* = 0.017 in Model 2 [TC, LDL-C, and eGFR were included as continuous variables]; HR = 1.80, 95% CI 1.05–3.10, *P* = 0.034 in Model 4 [TC, LDL-C, eGFR were included as categorical variables]) (Table 4).

**Table 4:** Multivariate Cox regression analysis of bone fracture on incident myocardial infarction in 2009–2015 merging the blood biomarker data

	2009–2015		
	HR	95%CI	*P* value
Model 1	1.88	1.10–3.24	0.022
Model 2	1.94	1.12–3.33	0.017
Model 3	1.78	1.03–3.06	0.038
Model 4	1.80	1.05–3.10	0.034

Model 1: adjusted for age, gender, BMI, hypertension, diabetes, smoking, alcohol consumption, TC, LDL-C (TC and LDL-C were included as continuous variables)

Model 2: Model 1 + eGFR (as continuous variables)

Model 3: adjusted for age, gender, BMI, hypertension, diabetes, smoking, alcohol consumption, TC, LDL-C (TC and LDL-C were included as categorical variables: for TC, < 5.2 and ≥ 5.2mmol/L, for LDL-C, < 3.4 and ≥ 3.4 mmol/L)

Model 4: Model 3 + eGFR (as categorical variables: <90 and ≥ 90 ml/min/1.73m^2^)

TC, total cholesterol; LDL-C, low density lipoprotein cholesterol; eGFR, estimated glomerular filtration rate

In the analysis of covariates on incident myocardial infarction, an elevated risk of incident myocardial infarction was also associated with higher age (HR = 1.09, 95% CI 1.08–1.10, *P* < 0.001) and higher BMI (HR = 1.05, 95% CI 1.03–1.07, *P* < 0.001). A history of hypertension (HR = 1.59, 95% CI 1.22–2.09, *P* < 0.001) was also associated with an increased risk of incident myocardial infarction (Table 5).

**Table 5:** Univariate and multivariate Cox regression analysis of covariates on incident myocardial infarction

	Crude				Multivariate*		
	**HR**	**95%CI**	**P value**		**HR**	**95%CI**	**P value**
Age, every 1 year increase	1.09	1.08–1.10	< 0.001		1.09	1.08–1.10	< 0.001
Female (vs. male)	0.91	0.73–1.13	0.372		0.84	0.65–1.08	0.178
BMI, every 1 kg/m^2^ increase	1.04	1.02–1.05	< 0.001		1.05	1.03–1.07	< 0.001
Hypertension (vs. without hypertension)	3.93	3.08–5.01	< 0.001		1.59	1.22–2.09	< 0.001
Diabetes (vs. without diabetes)	3.22	2.07–5.01	< 0.001		1.12	0.70–1.77	0.645
Smoker (vs. non-smoker)	1.36	1.08–1.72	0.009		1.02	0.74–1.41	0.901
Drinker (vs. non-drinker)	1.26	1.01–1.57	0.044		0.98	0.73–1.31	0.877

*Multivariate model included fracture, age, gender, BMI, hypertension, diabetes, smoking (ever), alcohol consumption (ever). BMI, body mass index

## Discussion

The present study has provided several noteworthy insights. Firstly, it showed that a history of bone fracture was significantly associated with an increased risk of myocardial infarction incidence. Secondly, the significant association between fractures and myocardial infarction was observed in the subjects with age ≥ 50 years but not in the subjects with age < 50 years, suggesting that aging plays a pivotal role in the association between fractures and myocardial infarction.

The incidence and estimated annual occurrence of myocardial infarction in this study are consistent with the findings of previous research. In a Danish population [3], over a 17-year period (2005–2021), 116,481 patients experienced their first acute myocardial infarction out of a total population of 4,202,034 to 4,687,295 individuals aged 18 years and older. This indicates a myocardial infarction incidence of 2.5–2.8% over 17 years, which is akin to the 2.5% incidence over 18 years (1997–2015) in this study. These earlier studies [1, 2] also demonstrated a declining trend in myocardial infarction rates, mirroring the present study, with an increasing emphasis on reducing risk factors at both the individual and community levels likely being the primary cause. Studies conducted in Denmark, northern Tanzania, the USA, Europe, and China have also projected similar incidences of myocardial infarction, ranging from 1.5 to 2.3 per 1,000 person-years [1–5].

To identify the association between fractures and incident myocardial infarction, we performed overall and subgroup analysis. The results showed that fractures were significantly associated with incident myocardial infarction, especially in the elderly subjects (age ≥ 50 years). Notably, osteoporotic fracture risk increases with age and is commonly observed in individuals aged over 50 years. The present study indicated that fractures were significantly associated with incident myocardial infarction in subjects more likely to develop osteoporosis. Fractures in younger individuals are rarely caused by age-related osteoporosis. These findings may explain the significant association observed between fractures and myocardial infarction only in the subjects with age ≥ 50 years. Age is the most important confounding factor in the present study. Aging may induce bone loss from normal skeletal system and anomalous deposition in the extra-skeletal system, which usually presents as osteoporotic fractures and heterotopic ossification [16]. Arterial calcification is a form of heterotopic ossification [28]. Arterial calcification may induce plaque instability, which leads to malignant cardiovascular events, such as myocardial infarction [9]. Aging is likely the link between fractures and myocardial infarction.

In a previous case-control study of 8,758 patients diagnosed with hip fractures matched with 35,032 controls without hip fractures [22], hip fractures were independently associated with a higher risk of subsequent acute myocardial infarction during a median 3.2-year follow-up (interquartile range 1.4–5.8 years). However, the myocardial infarction incidence in this study (6.82–8.7 per 1,000 person-years) was significantly higher than in previous studies, suggesting the inclusion of a high-risk population. A retrospective study [23] demonstrated that vertebral fractures (but not femoral or fractures in other sites) were correlated with myocardial infarction in hemodialysis patients but not in pre-dialysis patients with chronic kidney disease (also in a high-risk population). In the present study, within a natural population, we showed that fractures might be associated with an increased risk of incident myocardial infarction in the elderly population during long-term follow-up.

The present study had several limitations. Firstly, the absence of bone mineral density (BMD) or other related data in the CHNS dataset makes it challenging to establish a direct link between osteoporosis and the connection between bone fracture and myocardial infarction. This connection can only be inferred from the findings of other studies. Secondly, in our natural population, the incidence of myocardial infarction was relatively low. As a result, we were unable to conduct age-subgroup analyses in more detail ways.

In conclusion, based on our results and a review of the literature, it appears that the association between fractures and the risk of developing myocardial infarction is more likely to be significant in high-risk populations, particularly in elderly individuals or those with chronic kidney disease. Both aging and chronic kidney disease are recognized risk factors for osteoporosis. Intriguingly, glucocorticoids, another strong risk factor for osteoporosis [29], may also increase the risk of myocardial infarction [30]. These findings have the potential to contribute to the development and refinement of relevant theories.

## Data Availability

All data generated or analyzed during this study are included in this published article.
